# The colour stability of crystallized acetyl resin material in comparison to other restorative materials. An in-vitro study

**DOI:** 10.1038/s41405-020-00055-0

**Published:** 2021-02-24

**Authors:** Sarah Aburaisi, Abdulkarim Basha, Khalid Al Najjar, Husam Al Saqat, Faisal Al Askar, Fareed Al Nazer

**Affiliations:** 1 Department of Prosthodontics, College of Dentistry, Riyadh Elm University, Riyadh, 11681 Saudi Arabia; 2 Postgraduate Student, Preventive Department, College of Dentistry, REU, Riyadh, 11681 Saudi Arabia; 3 General Practioner, Private Practice, Riyadh, Saudi Arabia

**Keywords:** Fixed prosthodontics, Dental biomaterials

## Abstract

**Aim:**

To investigate the colour stability of Crystalized Acetyl Resin, in comparison to three other aesthetic restorative materials, when immersed in different staining aqueous solutions.

**Materials and methods:**

Fifteen disc-shaped specimens each, of the four materials tested, were immersed in either DW or 4 staining solutions. CIE L*a*b* values were recorded using a digital spectrophotometer on a weekly basis for 4 weeks. Data were compared using three- way/two-way ANOVA along with auto-regressive model.

**Results:**

Two-way Anova indicates that there is a significant colour change in all materials over time when immersed in the different staining solutions. Auto-regressive model showed that the colour change in each successive week was statistically significant (*P* < 0.001).

**Conclusion:**

The most staining solution affecting the materials during this study was black coffee. Within the limitation of this study, Acetyl Resin was the most colour stable material among the other materials.

## Introduction

Modern day patients seeking dental treatment are increasingly aware of the ever-evolving aesthetic options available. The wide variety of restorative materials available nowadays gives the dentists an advantage in performing the best possible treatments, while still maintaining the aesthetic requirements of the patient. Materials like Glass ionomer (GIC), Composite, and Bis-acryl are available to serve such purposes. Patients’ ever increasing demand for aesthetics, even for temporary restorations, obligates the dentists to ensure that they avail such restorative materials that can withstand changes in the oral environment and also must maintain their aesthetic appearance over the period of service.

Certain patients show reluctance towards the conventional options, possibly owing to insufficient time or funds. This obligates the dentists to seek-out and offer newer cosmetic treatment options that are non-invasive. As of today, some of these cosmetic treatments are fabricated from a synthetic polymer known as Crystalized Acetyl Resin, which is a thermoplastic material. Crystalized Acetyl Resin, also known as Polyoxymethylene (POM) or Acetal, is a chain of alternating methyl groups linked by an oxygen molecule.^1^ The crystalline structure provides the material with high strength, wear resistance and flexibility. This along with its high creep resistance, fatigue endurance, and hydrophobic property, makes it a promising material in the dental aesthetics field. It can be formed into homopolymers or a copolymer.^2^ Clinical uses of Acetal include: (1) RPD frameworks, enhancing the aesthetics and can be an alternative material for Co–Cr allergic patients.^2,3^ (2) Cosmetic smile enhancement. (3) Temporary restorations over Implants. (4) raising vertical dimension.^4^

Whenever a material is newly introduced for commercial purposes, and its prime objective is to obtain a superior aesthetic outcome, its colour stability is considered a very critical property, as it directly affects its clinical longevity and success.^5^ this warrants the comparison of its optical properties with other existing materials that are used for similar purposes. The emergence of the shade specific GIC material, in contrast to conventional GIC (white opaque), improved their used as a tooth-coloured restoration that could be used in aesthetic regions. Micro-hybrid filled composite resin, in contrast to conventional composite resin with different filler size, or amalgam, made it possible to perform temporary restorations and procedures of caries control and core build-up, while accounting for the aesthetic component of the restoration.^6^ It is known that filler types for Composite Resin have a deleterious effect on its stability in different aspects of its properties^7^ and not only limited to its colour stability. Bis-acryl is yet another excellent example of a material that is reported to be of superior colour stability when compared with other materials in its category of usage.^8^

While assessing colour changes, visual inspection is a subjective physiological and psychological process, while the spectrophotometer, when used as a colour evaluation device, not only eliminates subjective interpretations but also enables the identification of small colour differences. The CIE (Commission Internationale de l’Eclairage) L*a*b* colour system is a uniform colour scale that covers all the colours visible to the human eye and is thus suitable for perceptual studies of colour differences in dental materials.^9^ Using the CIE L*a*b* colour system, different thresholds of colour change (∆*E*) values that produce visually perceptible differences have been reported in several studies. Generally, a colour difference (∆*E*) value of 3.7 or more is considered as visually perceptible and clinically unacceptable.^10–12^

To achieve an accumulative colour change in a material and reach distinct results, most studies recommend the immersion to last for at least 4 weeks.^8,13–15^ Selection of the staining solution came according to drinks commonly used by a wide range of the population.^8,13,16^ which included coffee, tea and soda (S).

Since the clinical implication of studies affecting the colour stability of a material is mandatory, we found that it has been reported that two cups of coffee, tea, and/or soda are consumed every day; where they remain in contact with the materials in the oral environment for 10 min/day. With this said, 7 days of materials’ immersion into such solutions would account for 2.8 years of clinical life.^17^

The aims of this study were to determine the colour stability of crystallized acetyl resin in comparison to three others, commonly used, restorative materials when exposed to different staining liquids; and to determine a time-dependent correlation of any recorded colour change.

The null hypothesis is that there will be no difference in colour stability of crystalized acetyl resin when compared to GIC, composite resin, and bis-acryl resin.

## Materials and methods

### Materials

#### Armamentarium

**Fig. 1:**
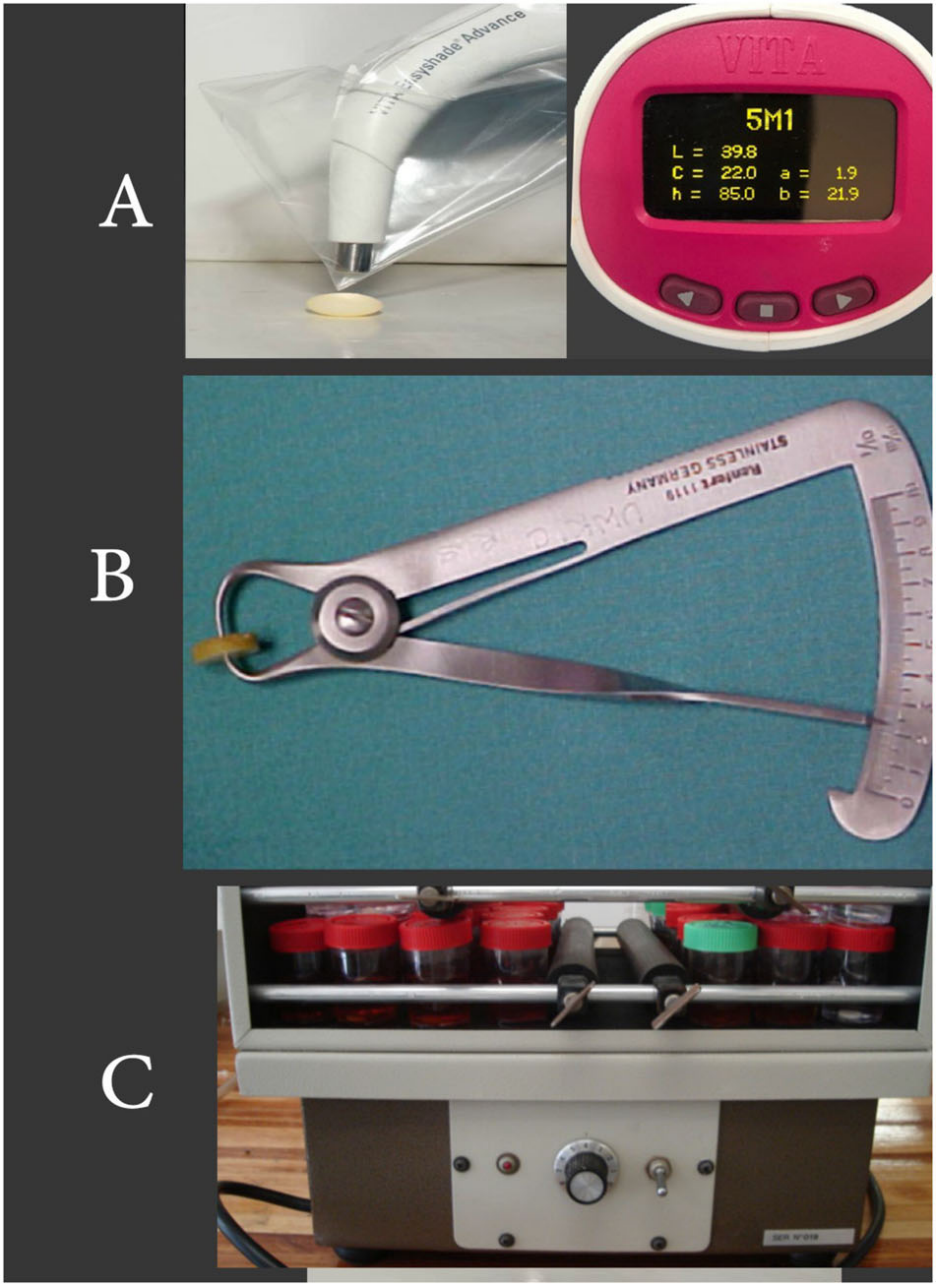
The shade. **A** Recording with spectrophotometer display against white background and L* a* b* readings. **B** Iwanson spring. **C** Orbital shaker.

**Fig. 2:**
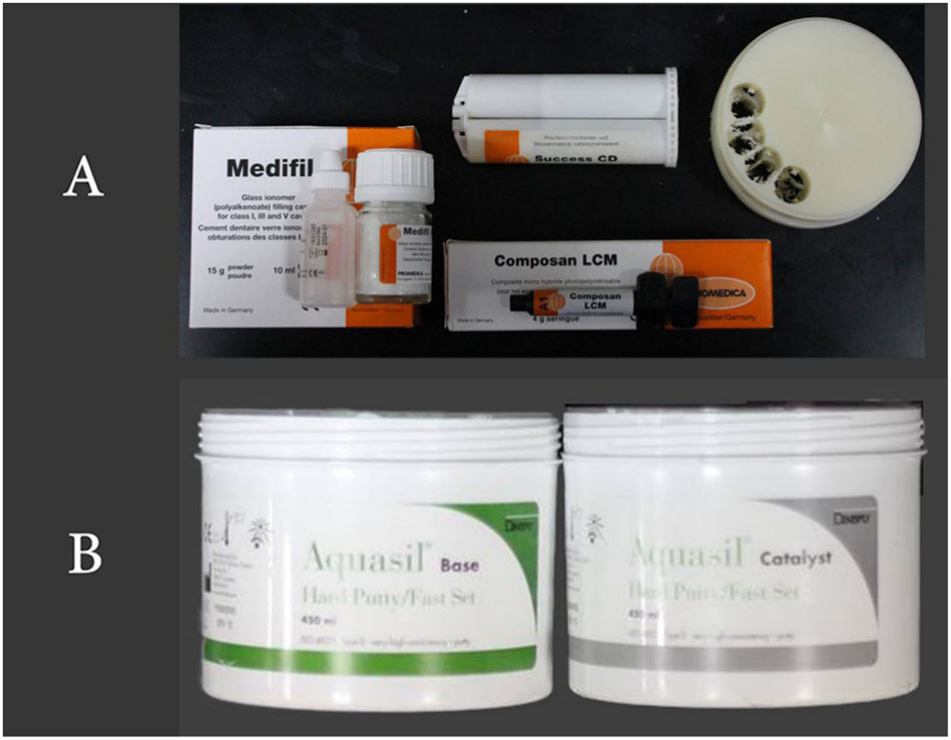
Materials used. **A** Tested materials. **B** Addition silicone.

The list of test materials, test solutions, auxiliary materials, instruments, and equipment are depicted in Table 1 and Figs. 1 and 2.

**Table 1:** Materials, instruments, and equipment used in the study.

**a. Test materials**				
**Material**	**Brand name**	**Manufacturer**	**Shade**	
Micro-Hybrid packable composite	Composan LCM micro-hybrid	Promedica, Germany	A1	
GIC	Medifil	Promedica, Germany	A1	
Bis-Acryl	Success CD	Promedica, Germany	A1	
Acetal	Acetal	Zirlux, Germany	A1	
**b. Test solutions**				
**Solution (Code)**	**Brand name**	**Manufacturer**	**Type**	**Group**
Distilled water (DW)	Eco water	Eco care LLC	Control	A
Black tea (BT)	Lipton Yellow Label tea	Unilever Mashreq -Tea Company, Egypt	Staining	B
Black coffee (BC)	Nescafe classic	Nestle, Egypt	Staining	C
Soda (S)	Pepsi	SSFCL Co., KSA	Staining	D
Energy drink (ED)	Code Red	Al Esayi Beverage Co. LTD., Jeddah, KSA	Staining	E
**c. Auxiliary materials**				
**Material**	**Brand name**	**Manufacturer**		
Poly vinyl siloxane	Aquasil putty	Dentsply Sirona, NY, USA		
**d. Instruments**				
**Instrument**	**Brand name**	**Manufacturer**		
Spatula	675-3 Cement Spatula	Medsey, Italy		
Cellophane sheet	N/A	Jarir Bookstore, KSA		
Iwanson caliper	1119 Stainless Steel Iwanson Caliper	Renfert, Germany		
Dispenser (Gun)	Garant™ Dispenser 1:1/2:1, 77580	3MESPE, St Paul, Minn, USA		
Glass stirrer	Glass rod	Ank trade store,Riyadh Saudi Arabia		
Tissue paper	Kleenex	Saudi paper company, Riyadh Saudi Arabia		
**e. Equipment**				
**Equipment**	**Brand name**	**Manfacturer**		
Curing light	Curing Light XL 3000	3MESPE, St Paul, Minn, USA		
Orbital shaker	Orbital Shaker Table CT-155	Cientec Laboratories Equipment, Piracicaba, São Paulo, Brazil		
Shade determinator	VITA Easyshade^®^ V Compact Advance 4.0	VITA Zahnfabrik H. Rauter GmbH & Co.KG, USA		
CAD/CAM	Inlab MC XL	Dentsply Sirona, NY, USA		
Glass slab	N/A	(Henry Schein co; New York, United States)

According to several studies, the lower the chroma of a material, the more markedly discoloured it becomes when compared to its more chromatic counterpart.^18–20^ taking this into consideration, shade A1 was selected for all the test materials.

#### Sample dimensions

Disc-shaped specimens of the four materials to be tested (Table 1a) were fabricated as stipulated by ISO #7491.^21^ with a diameter of 17 mm and thickness of 1 mm.

#### Sample size

In this study, a total of 15 specimens per test material (Total of 60) were fabricated.

### Methods

#### Specimen fabrication

##### Fabrication of CAD/CAM discs (crystalized acetyl resin)

The dimensions of the disc specimens were fed into the software (inLab CAM software, Sirona Co; New York, USA) to conform to the ISO #7491. Milling was done on a Crystalized Acetyl Resin block (Henry Schein co; Zirlux Acetal, Germany) using a (inLab MC XL, Sirona Co; New York, USA). After milling of the specimens all the specimens were measured to ensure uniform thickness and diameter using an Iwanson spring caliper (Renfert, Germany, 1119 stainless).

##### Silicone mould fabrication

**Fig. 3:**
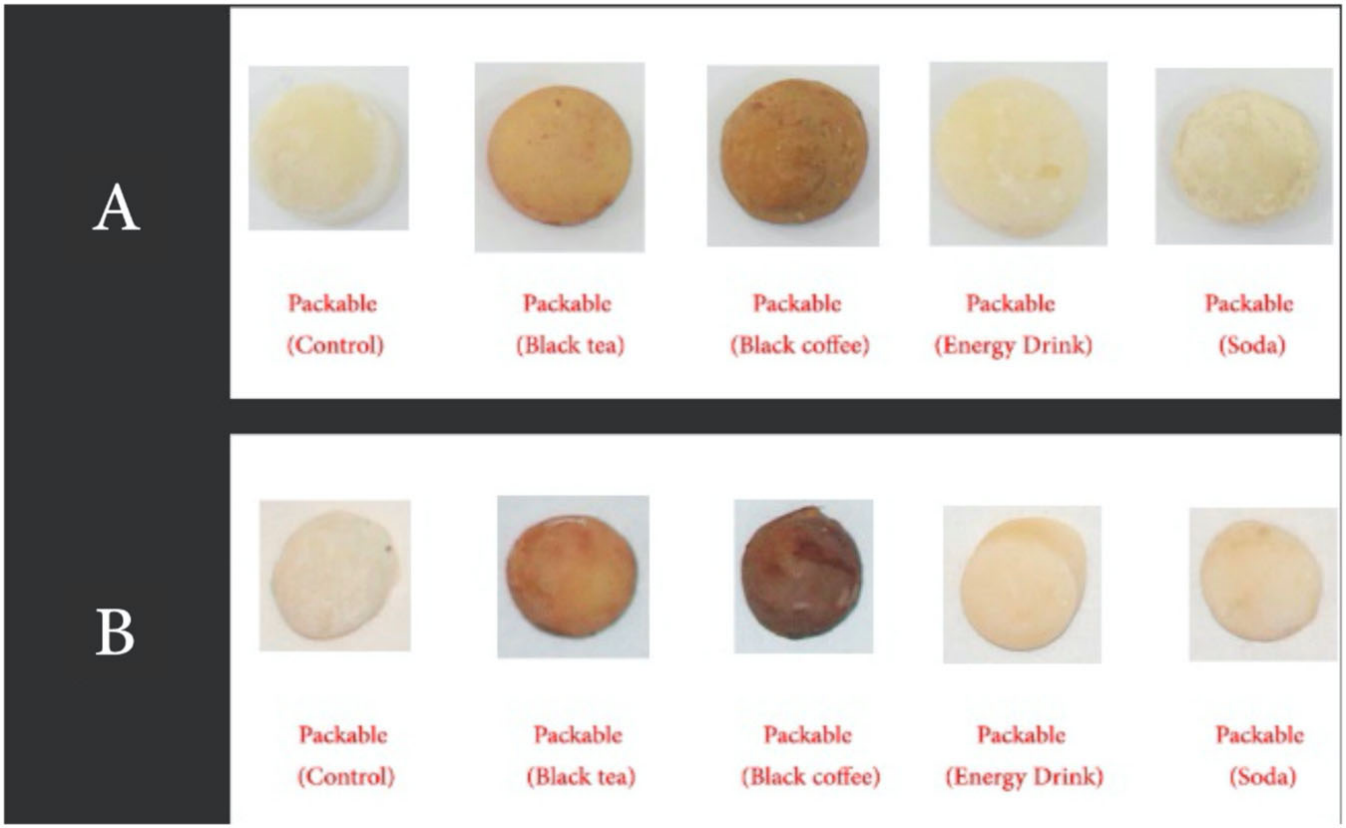
Composite Speciments ready for measurements. **A** Composite resin (after 2 weeks). **B** Compositeresin (after 4 weeks).

**Fig. 4:**
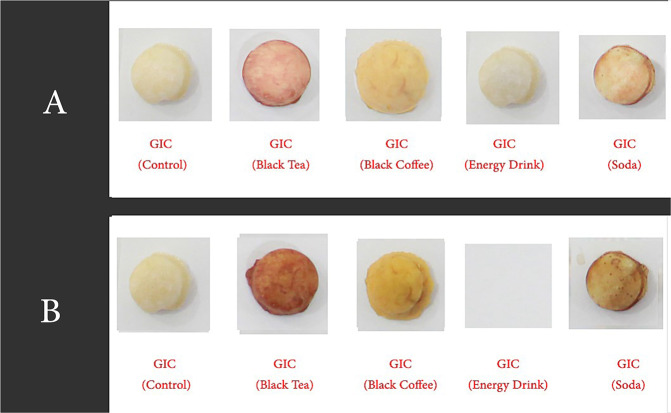
GIC Speciments ready for measurements. **A** GIC (after 2 weeks). **B** GIC (after 4 weeks).

**Fig. 5:**
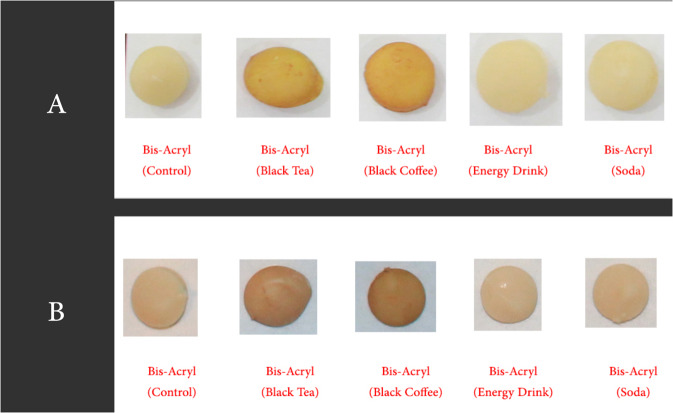
Bis-acryl Speciments ready for measurements. **A** Bis-acryl (after 2 weeks). **B** Bis-acryl (after 4 weeks).

**Fig. 6:**
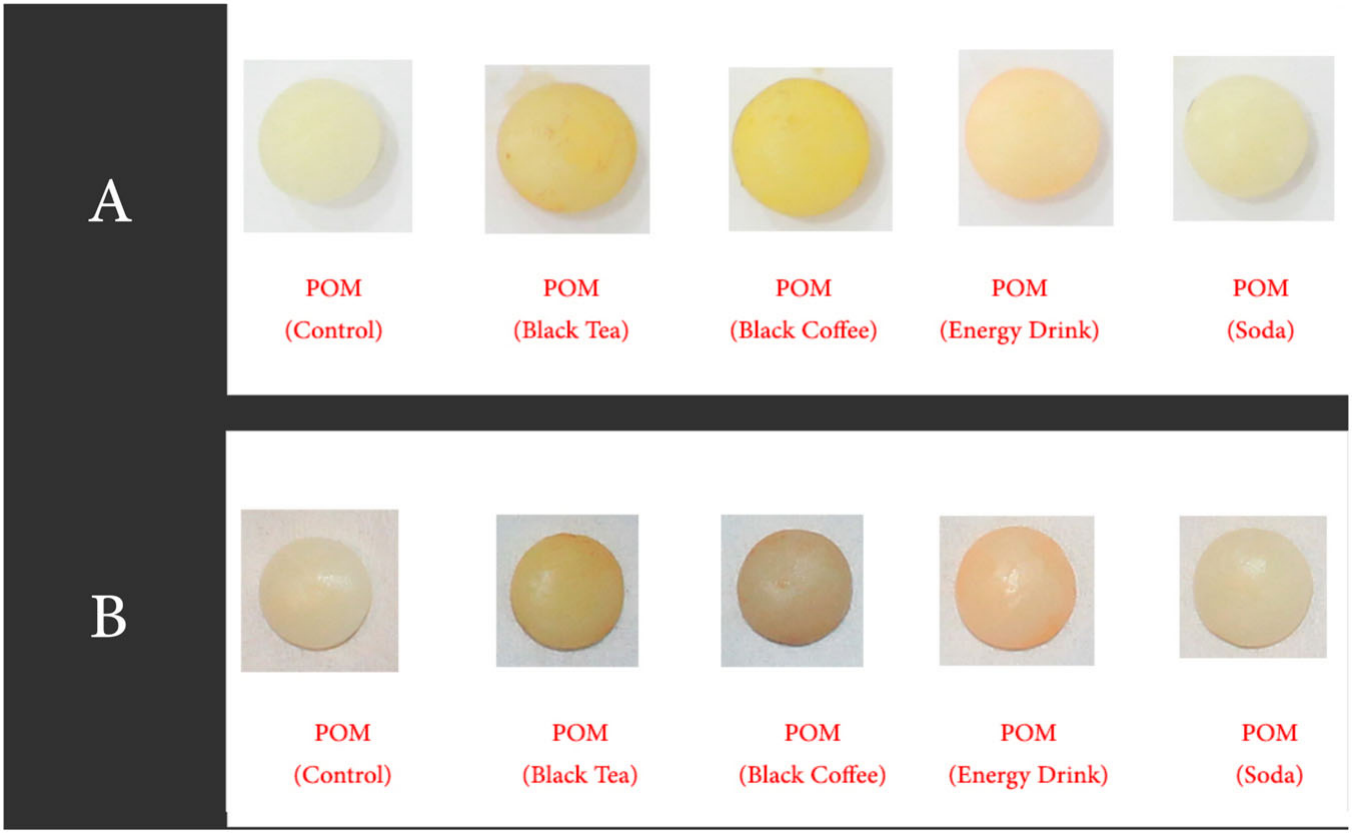
POM Speciments ready for measurements. **A** POM (after 2 weeks). **B** POM (after 4 weeks).

For fabrication of discs that did not involve the CAD/CAM technique, a Silicone mould was fabricated to duplicate the disc made by CAD/CAM. A plastic container with dimensions of 9 cm width, 17 cm length, and 10 mm height was used. The polyvinylsiloxane PVS (Aquasil Putty, Sirona co; New York, USA) (Fig. 2B) was mixed according to the manufacturer’s instructions and inserted inside the plastic box. One of the discs fabricated using CAD/CAM were placed over a glass slab and the plastic box containing the putty material was gently but firmly inverted over it till the edges of the box contacted the glass slab. The excess material was cut off and the entire ensemble was held in place till the PVS material had set. The disc was removed from the PVS material rendering a mould space for the fabrication of the remaining specimens (Figs. 3–6).

##### Fabrication of packable composite discs

Packable composite (Promedica co; Composan LCM, Neumunster, Germany) was dispensed onto a spatula and introduced into the silicone mould till filled. A cellophane sheet was placed over the material and thereafter pressed down using a glass slab, to ensure uniform thickness as well as a smooth surface. The material was light-polymerized through the glass slab using Curing Light XL 3000 with an intensity of 400 mW/cm^2^ (3MESPE, St Paul, Minn) for 20 s with the light tip ~1 mm away from the specimens.^22^ upon polymerization, the specimens were removed from the mould and examined visually for any porosities. The consistency of the testing surface was also observed to assess complete polymerization. Any specimen with fabrication errors was excluded and another specimen was fabricated in its place. All the specimens were measured to ensure uniform thickness and diameter using an Iwanson spring caliper (Renfert, Germany, 1119 stainless).

##### Fabrication of GIC discs

GIC (Medifil, Promedica co; Neumünster, Germany) was dispensed onto a mixing pad and mixed according to the manufacturer’s instructions. The material was then introduced into the silicone mould till filled. A cellophane sheet was placed over the material and thereafter pressed down using a glass slab, to ensure uniform thickness as well as a smooth surface. After the specified setting time had elapsed, the specimens were removed from the mould and examined visually for any defects. Any defective specimens were discarded and replaced with a freshly fabricated one. All the specimens were measured to ensure uniform thickness and diameter using an Iwanson spring (Renfert, Germany, 1119 stainless).

##### Fabrication of bis-acrylic discs

Bis-acrylic (Success CD, Promedica co; Neumunster, Germany) was dispensed directly into the mould space from the cartridge through the dispensing tips provided by the manufacturer. A cellophane sheet was placed over the material and thereafter pressed down using a glass slab, to ensure uniform thickness as well as a smooth surface. After the specified setting time had elapsed, the specimens were removed from the mould and examined visually for any defects. Any defective specimens were discarded and replaced with a freshly fabricated one. All the specimens were measured to ensure uniform thickness and diameter using an Iwanson spring caliper (Renfert, Germany, 1119 stainless).

#### Specimen pre-treatment

All the test specimens, from the four materials, were rinsed thoroughly with distilled water (DW) to remove any debris. A total of 60 coded plastic containers were used to store the specimens in DW at 37 ± 1 °C for 24 h to stimulate the condition of oral cavity environment.^23^

#### Preparation of staining solutions

Four staining agents were used, namely, black tea (BT), black coffee (BC), soda, and energy drink (ED); and the control solution was DW (Table 1b). The selected staining solutions were identified for the study as they have been commonly used by several researchers.^8,13,14^

The BT solution was prepared by immersing five teabags (Lipton Yellow Label Tea, Unilever Mashreq -Tea Company, Egypt) into 1000 ml of boiled water. The BC solution contained 20 g of coffee (Nescafe Classic, Nestle Egypt) poured into 1000 ml of boiled water. Both solutions were stirred every 30 min for 10 s until they cooled down to room temperature (25 °C) and were then filtered through a filter paper.^24^

No preparation was required pertaining to the soda, ED, and DW solutions.

During the duration of the study, the solutions were renewed every 2 days.^25^

#### Immersion of specimens in aqueous solutions

After 24 h in DW, the 15 specimens of each material were randomly divided into five groups (*n* = 3)^26^ and based on the test solution they were immersed in, they were named group A, B, C, D, and E (Table 1b). To prevent air entrapment and sedimentation of the solutions over the specimens, an Orbital shaker (Orbital Shaker Table CT-155, Cientec Laboratories Equipment, Piracicaba, São Paulo, Brazil) (Fig. 1C), was used in cycles. Each cycle comprised of using the orbital shaker 4 times a day, at 30 revolutions per minute, and with 1-h intervals.^15,25^ Such cycles were repeated every 24 h over the course of the study.

#### Cleaning procedure before recording colour measurements

At each time interval of the experiment, prior to recording the colour measurements, all the specimens were removed from their respective solutions, rinsed with DW for 5 min, and blotted dry with tissue paper. Any accumulated surface sediment was thus removed. Once the specimens had completely dried, colour measurements were taken on the testing side of each specimen disc. Thereafter, the specimens were re-immersed in their respective storage solutions.^22,25^

#### Colour measurement

The timeline observed for measurements were as follows: (1) at baseline, i.e., after 24 h in DW (T0), (2) after 7 days (T1), (3) after 2 weeks (T2), (4) After 3 weeks (T3), and after 4 weeks (T4).

The colour or shade measurements were recorded for all the specimens against a white background using a digital spectrophotometer (VITA Easyshade^®^ V Compact Advance 4.0, VITA Zahnfabrik H. Rauter GmbH & Co.KG or shade)^26–28^ (Fig. 1A).

After obtaining the colour and shades measurements, each specimen was placed in its correspondingly labelled plastic container containing the respective control or staining agent.

Colour and shade measurements were recorded according to their L*, a*, and b* values, where L* indicates brightness or lightness (value), a* indicates the colour on the red-green coordinate, and b* indicates the colour on the yellow–blue coordinate.^29^ An overall shade was obtained upon every measurement (T0, T1, T2, T3, and T4) (Fig. 1A).

#### Determination of colour and calculation of colour changes

The colour and the shade change of each specimen were measured every week (1, 2, 3, and 4) relative to baseline shade, and the data of colour and shade change (ΔL*, Δa*, Δb*) measurements were entered onto an excel spreadsheet. The total colour change/difference (∆E*) was calculated for each specimen relative to its baseline colour using the colour difference formula: ΔE* = [(ΔL*)^2^ + (Δa*)^2^ + (Δb*) ^2^]^1/2^.^30^

#### Data tabulation and analyses

All data were entered into an excel spreadsheet. The colour changes in ΔL*, Δa*, Δb* and total colour changes [ΔE*(T4−T0)] for all the intervals were calculated using an Excel computer software package (Excel 7.0; Microsoft, Redmond, Wash).

The data were analyzed using Statistical Package for the Social Sciences Version 24 (IBM, USA). Discolouration results were analyzed by *A three-factor analysis of variance (ANOVA)* (with repeated measures on one factor (time) to investigate if statistically significant differences (*P* < 0.001) existed between the experimental groups across time. *Two-way ANOVA* was carried out to test if statistically significant (*P* < 0.001) existed between the experimental groups at each week, and *auto-regressive model* was done to account for the rate of change in colour as the weeks progressed.

## Results

### Repeated measures ANOVA for three factors

The results indicate that the effect of all three factors, i.e., material, solution, and duration of time, are statistically significant. The effects of various solutions on the various materials also showed statistical significance (Table 2).

**Table 2:** Three-way analysis of variance.

Effect	DF	*F* value	Pr > *F*
Material	3	27.80	<0.0001
Solution	4	120.07	<0.0001
Time	3	28.78	<0.0001
Material × Solution	12	3.94	<0.0001
Material × Time	9	0.90	0.5300
Solution × Time	12	2.63	0.0030
Material × Solution × Time	36	1.05	0.4080

### Repeated measures ANOVA for two factors

The results indicate that there is a statistical significance of the effects of the solution on the material at each time interval (per week) (Table 3).

**Table 3:** Two-way analysis of variance.

Effect	DF	*F* value	Pr > *F*
Week 1			
Materials	3	6.76	0.001
Solutions	4	14.75	<0.001
Materials × Solutions	12	2.00	0.051
Week 2			
Materials	3	4.46	0.009
Solutions	4	25.91	<0.001
Materials × Solutions	12	0.98	0.487
Week 3			
Materials	3	5.33	0.003
Solutions	4	39.27	<0.001
Materials × Solutions	12	1.14	0.356
Week 4			
Materials	3	13.06	<0.001
Solutions	4	47.85	<0.001
Materials × Solutions	12	2.72	0.009

### Auto-regressive model

The results show that the colour changes for each material as time progressed, showed statistically significant differences to the preceding week (*P* < 0.001) (Table 4).

**Table 4:** Auto-regressive model.

Materials	Estimate	SE	*P*	95% CI	
ALL	0.53	0.06	<0.001	0.42	0.64
GIC only	0.58	0.11	<0.001	0.36	0.79
Bis-acryl only	0.45	0.12	<0.001	0.21	0.68
Packable only	0.49	0.11	<0.001	0.27	0.72
POM only	0.50	0.11	<0.001	0.27	0.72

## Discussion

One of the most important aesthetic requirements of a restorative material to prove its longevity is its colour stability, especially when exposed to different oral environmental conditions.^8^ The current research evaluated the colour stability of four commonly used aesthetic restorative materials, with the aim of assessing if crystalized acetyl resin showed superior colour stability over time when immersed in four staining solutions.

Through the results of the study, it was revealed that the colour changes seen in crystalized acetyl resin showed statistical significance, as was the case with the other restorative materials tested, and it was significantly influenced by the time and staining solution. In light of these findings, the null hypothesis was rejected.

It was seen that BT and BC had the highest chromogenic properties which is in agreement with the results found in a previous study, where they concluded that the staining solution was the most significant factor affecting the colour stability.^30^

**Fig. 7:**
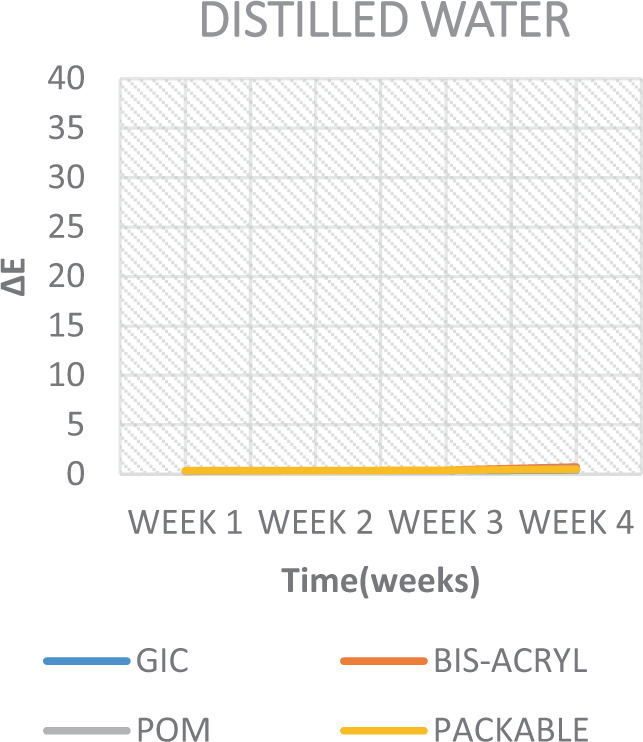
Colour change over time of test materials in distilled water.

It was also seen that the least effective solution for discolouring the test materials across the board, was DW, wherein it showed no perceptible colour changes (∆*E* < 3.7) (Table 5 and Fig. 7). This was concurrent with a similar study and justifies its use as a control solution.^31^

**Table 5:** Weekly ∆*E* values.

	Week 1	Week 2	Week 3	Week 4
GIC (control)	0.31 (0.06)	0.34 (0.19)	0.38 (0.20)	0.42 (0.21)
GIC (black coffee)	13.50 (3.03)	13.48 (1.83)	16.14 (5.23)	17.61 (2.90)
GIC (black tea)	9.57 (1.43)	13.21(3.11)	18.10 (2.03)	28.08 (5.92)
GIC (soda)	4.75 (2.82)	9.75 (1.42)	12.49 (4.80)	17.71 (7.16)
GIC (energy drink)	1.37 (0.48)	8.89 (5.81)	6.94 (6.40)	0.00 (0.00)
Bis-acryl (control)	0.33 (0.12)	0.34 (0.29)	0.42 (0.29)	0.73 (0.43)
Bis-acryl (black coffee)	14.25 (3.25)	18.32 (7.30)	19.10 (3.58)	21.86 (6.73)
Bis-acryl (black tea)	7.97 (2.30)	11.30 (0.70)	19.57 (2.18)	19.85 (2.28)
Bis-acryl (soda)	10.91 (10.16)	14.00 (3.11)	18.11 (2.48)	21.29 (5.58)
Bis-acryl (energy drink)	5.92 (3.67)	6.57 (4.46)	7.23 (5.87)	10.92 (8.88)
POM (control)	0.28 (0.06)	0.34 (0.30)	0.36 (0.28)	0.54 (0.38)
POM (black coffee)	8.05 (0.91)	11.43 (4.41)	12.80 (4.41)	15.55 (4.41)
POM (black tea)	3.22 (0.70)	13.09 (4.81)	18.11 (5.33)	18.34 (3.47)
POM (soda)	3.38 (0.75)	5.84 (6.57)	9.90 (0.97)	11.51 (1.31)
POM (energy drink)	4.07 (1.16)	3.78 (1.98)	8.65 (7.26)	9.85 (7.94)
Composite (control)	0.36 (0.14)	0.37 (0.21)	0.42 (0.57)	0.47 (0.71)
Composite (black coffee)	17.86 (3.01)	17.45 (4.47)	27.58 (1.34)	36.05 (1.80)
Composite (black tea)	3.22 (0.70)	13.09 (4.81)	18.11 (5.33)	18.34 (3.47)
Composite (soda)	7.64 (4.92)	13.69 (7.89)	17.90 (2.56)	20.25 (2.43)
Composite (energy drink)	6.78 (1.79)	10.57 (4.08)	11.51 (6.04)	17.06 (2.85)

**Fig. 8:**
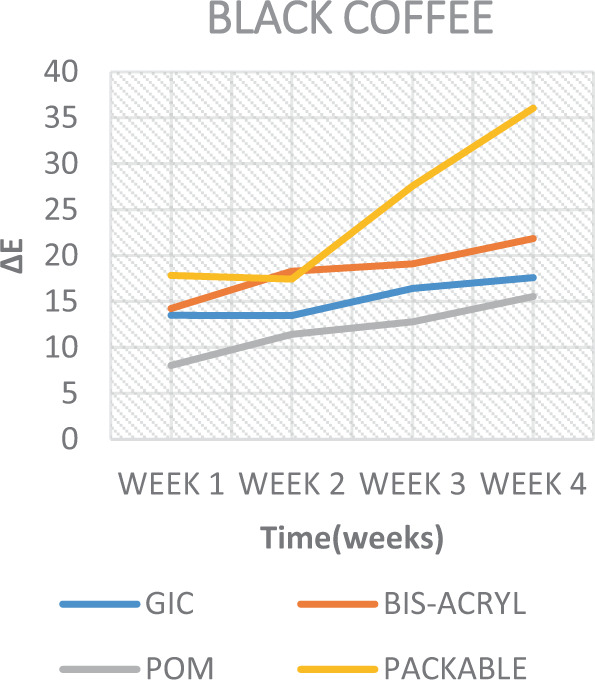
Colour change over time of test materials in black coffee.

**Fig. 9:**
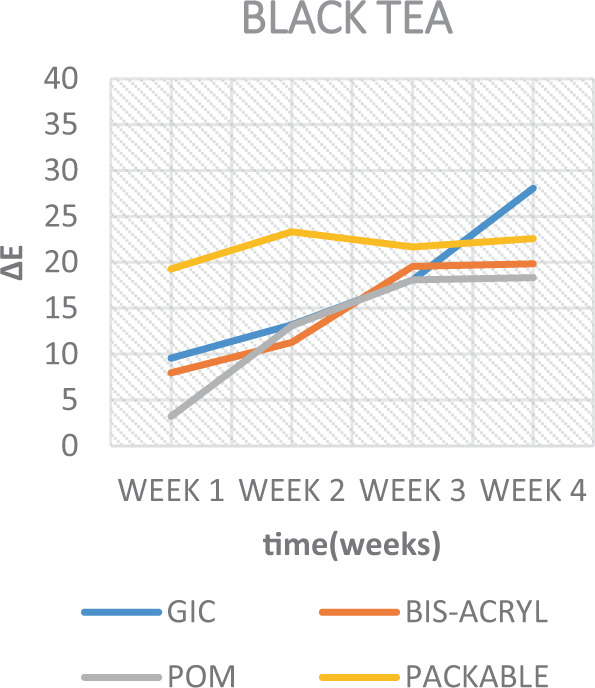
Colour change over time of test materials in black tea.

When analyzing the staining solutions, the results showed distinct effects depending on the material used. Overall, the solutions imparting the highest colour staining properties (Table 5) were BC (In composite (∆*E* 36.05)) (Fig. 8) and BT (In GIC (28.08)) (Fig. 9). The findings of this study is in agreement with the findings of Raeisosadat et al.,^32^ which suggested that BC affected the colour stability of composite more than BT and other solutions. While interpreting the results of staining solutions on GIC, the GIC was affected more by BT. These results is in agreement with the study of Hamid et al.,^17^ which indicated that BT affected the colour stability of GIC more than BC and other solutions. Whatever the case is, according to Sezai et al.^33^ and Um and Ruyter,^13^ such discoloration might be due to the adsorption and absorption process of the yellow colourant particles (Tannic acid) present in coffee; whereas for tea, it might be due to the adsorption process alone. These two processes are further explained to be due to the high affinity of the polymer phase of the materials to the colourant particles.^13^

**Fig. 10:**
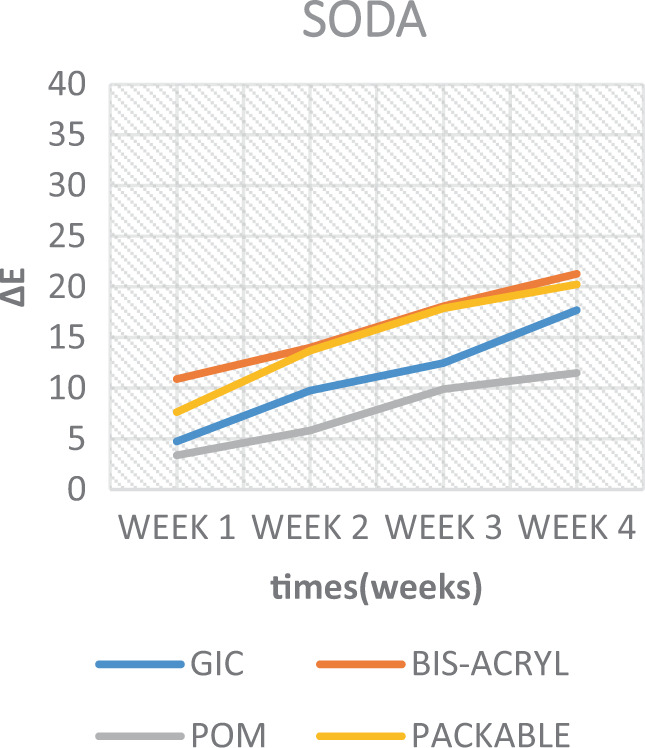
Colour change over time of test materials in soda.

**Fig. 11:**
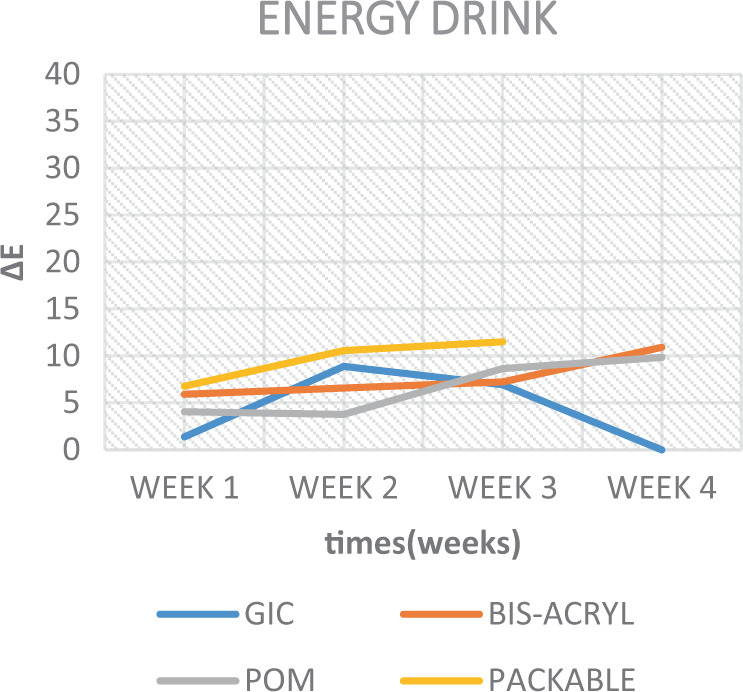
Colour change over time of test materials in energy drink.

When inspecting the results of the soda and the ED, less colour change was observed in the samples (Table 5, Figs. 10 and 11). Although, when considering such solutions, their low pH is considered to be a convincing factor for surface deterioration; thereby causing a dynamic colour change. However, this was not the case here. As Um and Ruyter.^13^ mentioned, this is possibly attributed to their lack of yellow colourants. However, when taking into consideration the surface integrity of the GIC material immersed in the soda and ED, a deterioration of the surface was seen in soda, while an unanticipated full degradation of the discs was observed in ED. A similar deterioration is interpreted in another study,^34^ and was attributed to the presence of acids in such drinks. These, exceptionally low pH solutions by nature, caused the chelation of cement-forming ions, such as calcium, to an insoluble product.^24^

Packable composite showed the highest total colour change (∆*E* = 36.05) (Table 5). Composite, as a material, is made of a resin matrix, fillers, and silane coupling agent. The ability of solutions to cause staining in the composite material has been reported to be due to the absorption of water particles into the resin matrix.^35^ Composite resins that can absorb water are also able to absorb other fluids and is attributed to the hydrophilicity of the resin matrix.^35^ The water particles and/or fluids, when absorbed, causes expansion and plasticizing of the resin matrix; consequently causing micro-cracks and weaker bonds between the constituents (Fillers and Resin matrix).^36^ This allows for the penetration of the stains. This is applicable to all the solutions in this study. Although, soda, and ED might have a supplemental erosive effect, the absence of yellow colourants in them, as mentioned previously, made them less effective.^13^

Regarding the GIC material, the method of staining is explained by many studies to be a result of degradation of metal polyacrylate salts in GIC.^37^ This subsequently results in a rougher surface with voids, and the undissolved glass particles result in greater water and food colourant absorption.^38^ Thus, the staining of the GIC material was caused by the porosity of the glass particles, and micro-cracks.^39^ This process was also supported by another study, which concluded that a rougher surface of the GIC would show more colour change than its smoother counterpart.^40^

When assessing the results of the bis-acryl, colour change was observed in all staining solutions like all the other materials, with BC showing the highest change (∆*E* = 21.86) (Table 5). The colour change in this specific material, which is a resin based material, is attributed to the same factors affecting composite resin.^41^ In addition, it has also been reported that the discolouration be attributed to the alteration or oxidation of the amine accelerators in the material.^13^

The discoloration of crystalized acetyl resin has also been attributed to the properties of adsorption and absorption of water particles, as is the case with composite resin. Arikan et al.;^42^ measured the water sorption of acetal material and reported that the material had acceptable water sorption according to ISO specification. Regardless of the amount of water sorption, the fact that a material absorbs water, renders it likely to absorb other fluids and would be accountable for the discolouration seen. Nevertheless, Acetal was considered to be the most colour stable among the other materials tested in this study. This might be due to its crystalline component and that it is monomer free; as monomers are regarded to be responsible for colour change in most resinous materials.^1^ The solution that discoloured POM the most, in this study, was BT (∆E = 18.34). This is in disagreement with what Hamid et al.^43^ found and this might be due to the difference in the fabrication of the staining solutions and/or not using an orbital shaker. The omission of the orbital shaker might have caused sedimentation of the solution over the specimen thereby affecting the colour measurements in their study.

The results of the current study also depicted that colour stability was affected over time and were subsequently rendered more stained after each successive time interval (Figs. 7–11). This is in concurrence with a similar study where they reported that, as time increases, the tooth-coloured restorations become more discoloured.^44^

## Conclusions

Within the limitations of this study, the conclusions are as follows:

1. Acetyl resin was the most colour stable material among the other materials.
2. Discolouration of acetyl resins increased proportionally with the immersion period.
3. The most chromogenic staining solution for acetal was found to be the BT; while BC exhibited the most effects in the remaining materials.
4. All the test materials showed clinically perceptible changes in colour with the four staining solutions.

### Limitations

1. Unexpected reaction of GIC in ED, which resulted in the full dissolution of the specimens by the end of the fourth week, rendering them unacceptable for providing readings.^36^
2. It have been described in the literature that there are different brands and types of the digital spectrophotometer. Some of which are superior in colour determination than the Vita Easyshade V. Thus, it was essential that we declare such downside of this device.^45^
